# cGAS–STING–NF-κB Axis Mediates Rotenone-Induced NLRP3 Inflammasome Activation Through Mitochondrial DNA Release

**DOI:** 10.3390/antiox14111276

**Published:** 2025-10-24

**Authors:** Yewon Mun, Juseo Kim, You-Jin Choi, Byung-Hoon Lee

**Affiliations:** 1 College of Pharmacy and Research Institute of Pharmaceutical Sciences, Seoul National University, Seoul 08826, Republic of Korea; 2 College of Pharmacy, Daegu Catholic University, Gyeongsan 38430, Republic of Korea

**Keywords:** rotenone, NLRP3 inflammasome, cGAS–STING pathway, mitochondrial DNA, NF-κB

## Abstract

Rotenone, a classical inhibitor of mitochondrial complex I, disrupts electron transport and promotes the generation of reactive oxygen species (ROS), contributing to inflammation and cell death. However, the precise molecular mechanisms linking mitochondrial dysfunction to inflammatory signaling remain incompletely understood. In this study, we investigated the role of the cGAS–STING pathway in rotenone-induced NLRP3 inflammasome activation in PMA-differentiated THP-1 macrophages. Rotenone treatment activated the cGAS–STING axis, as evidenced by increased cGAS expression and the phosphorylation of STING and TBK1. This activation led to the nuclear translocation of NF-κB and the upregulation of NLRP3, promoting inflammasome priming and IL-1β secretion. Inhibition of STING using H-151 markedly suppressed NLRP3 expression, NF-κB activation, and IL-1β release. Similarly, cyclosporin A, an inhibitor of mitochondrial permeability transition pore opening, reduced mitochondrial ROS, cytosolic oxidized mitochondrial DNA, and downstream activation of the cGAS–STING pathway, thereby attenuating inflammasome activation. These findings demonstrate that rotenone activates the NLRP3 inflammasome via mitochondrial ROS-mediated release of mtDNA and subsequent activation of the cGAS–STING–NF-κB signaling axis in THP-1-derived macrophages.

## 1. Introduction

The NLR family pyrin domain containing 3 (NLRP3) inflammasome is a crucial component of the innate immune system that detects intracellular stress and initiates inflammatory responses [1]. Upon activation, NLRP3 assembles into a multiprotein complex with the adaptor protein ASC, which in turn activates caspase-1 [2]. Activated caspase-1 cleaves the precursor forms of pro-inflammatory cytokines, such as pro-IL-1β and pro-IL-18, into their mature forms, IL-1β and IL-18, enabling their secretion and triggering inflammation [3]. In parallel, caspase-1 also cleaves gasdermin D (GSDMD), which forms pores in the plasma membrane, contributing to the release of inflammatory mediators and initiating pyroptotic cell death [4].

Activation of the NLRP3 inflammasome is tightly controlled and proceeds through two distinct steps: priming and activation. In resting cells, the expression levels of NLRP3, pro-IL-1β, and pro-IL-18 are low, whereas ASC and caspase-1 are constitutively expressed [5]. The priming step is essential to upregulate the transcription of NLRP3 and pro-inflammatory cytokines, enabling cells to mount a subsequent inflammasome response. This first signal is mediated by NF-κB–dependent transcription, triggered by Toll-like receptor (TLR) agonists or inflammatory cytokines such as TNF-α or IL-1β [5,6]. For example, lipopolysaccharide (LPS), a well-characterized priming agent, activates TLR4 signaling, leading to NF-κB nuclear translocation and induction of NLRP3 and its downstream effectors [5]. Following priming, a second signal initiates inflammasome assembly. Diverse stimuli such as extracellular ATP or the ionophore nigericin activate the inflammasome by inducing K^+^ efflux [7,8]. Although the precise mechanisms remain incompletely defined, K^+^ efflux has been shown to disrupt intracellular calcium homeostasis and promote mitochondrial dysfunction [9,10,11]. Damaged mitochondria generate excessive mitochondrial reactive oxygen species (ROS), which not only activate the NLRP3 inflammasome directly [12], but also oxidize mitochondrial DNA (mtDNA). Oxidized mtDNA is released into the cytoplasm through the mitochondrial permeability transition pore (mPTP), where it binds to and activates NLRP3 [13,14,15]. In addition, cardiolipin, a phospholipid typically confined to the mitochondrial inner membrane, can translocate to the outer membrane during mitochondrial stress and serve as an endogenous ligand for NLRP3 [16,17].

Beyond its role in directly engaging NLRP3, cytosolic oxidized mtDNA also functions as a damage-associated molecular pattern (DAMP) that activates other innate immune pathways including TLR9 and cyclic GMP–AMP synthase (cGAS) [18]. TLR9, primarily localized to endosomal compartments, senses endocytosed double-stranded DNA (dsDNA), including mtDNA, and triggers the NF-κB-mediated transcription of pro-inflammatory cytokines [19,20,21]. In contrast, cGAS acts as a cytosolic DNA sensor that is broadly expressed across cell types [22,23]. Upon binding to dsDNA, cGAS catalyzes the production of 2′3′-cyclic GMP–AMP (cGAMP), which in turn activates the adaptor protein STING on the endoplasmic reticulum membrane [24]. Activated STING oligomerizes and translocates to the Golgi, where it recruits TANK-binding kinase 1 (TBK1), leading to phosphorylation of IRF3 and IκB, and subsequent activation of type I interferon and NF-κB signaling. Emerging evidence suggests that the cGAS–STING axis not only promotes inflammatory cytokine production, but also plays a role in priming or amplifying NLRP3 inflammasome activation [25,26,27].

Rotenone is a naturally occurring mitochondrial complex I inhibitor that has been widely used as a pesticide and herbicide [28]. The compound’s toxicity has been recognized since the 1990s, and the World Health Organization classifies it as a Class II moderately hazardous pesticide. Acute exposure to rotenone can result in central nervous system abnormalities, while chronic exposure has been implicated in the development of neurodegenerative diseases such as Parkinson’s disease [29,30,31]. In addition to its neurotoxicity, rotenone has been associated with hepatotoxicity and nephrotoxicity, though the mechanisms remain incompletely understood [32,33]. At the cellular level, rotenone inhibits electron transfer in mitochondrial complex I, leading to disrupted electron transport, elevated mitochondrial ROS generation, and impaired ATP synthesis [34]. Several studies have demonstrated that rotenone can activate the NLRP3 inflammasome [35,36], but the upstream mitochondrial signals that link rotenone-induced dysfunction to inflammasome assembly, particularly the roles of mitochondrial ROS, oxidized mtDNA release, and cGAS–STING signaling, remain poorly characterized.

In this study, we explored whether rotenone activates the NLRP3 inflammasome in THP-1-derived macrophages via mitochondrial ROS–mediated release of oxidized mtDNA and subsequent activation of the cGAS–STING signaling pathway. Our findings provide new mechanistic insights into the inflammatory response induced by mitochondrial toxins and highlight the interplay between mitochondrial dysfunction, cytosolic DNA sensing, and inflammasome activation.

## 2. Materials and Methods

### 2.1. Cell Culture and Differentiation

THP-1 human monocyte cells (ATCC) were cultured in RPMI 1640 medium (Gibco, Grand Island, NY, USA; A1049101) supplemented with 10% fetal bovine serum (FBS; Gibco, 16000044) and 1% antibiotic-antimycotic solution (Gibco, 15250062) at 37 °C in 5% CO_2_. For differentiation into macrophage-like cells, THP-1 cells were seeded at a density of 1 × 10^6^ cells/mL and treated with 50 ng/mL phorbol 12-myristate-13-acetate (PMA; Sigma, St. Louis, MO, USA; P8139) for 24 h. The medium was then replaced with fresh RPMI 1640 without PMA, and cells were further incubated for 24 h before experiments. Bone marrow-derived macrophages (BMDMs) were isolated from mouse femurs and tibias as previously described [37]. Briefly, bone marrow cells were flushed with PBS and cultured in RPMI 1640 containing 10% FBS, 1% antibiotic-antimycotic solution, and 20 ng/mL M-CSF (Gibco, PMC2044) for 7 days to allow for macrophage differentiation. Adherent cells were used for experiments as mature BMDMs.

### 2.2. Western Blot Analysis

Cells were lysed in buffer containing 50 mM HEPES, 150 mM NaCl, 5 mM EGTA, 50 mM β-glycerophosphate, 1% Triton X-100, and protease inhibitor cocktail (Roche, Basel, Switzerland). Lysates were centrifuged at 14,000 rpm for 15 min at 4 °C, and supernatants were collected. Equal amounts of protein were resolved by SDS-PAGE (8–12%) and transferred onto PVDF membranes (Cytiva, Marlborough, MA, USA; 10600023). Membranes were blocked in 5% BSA/TBST and incubated with primary antibodies overnight at 4 °C. After incubation with HRP-conjugated secondary antibodies (Cell Signaling Technology, Danvers, MA, USA), bands were visualized using SuperSignal West Pico PLUS substrate (Thermo Scientific, Waltham, MA, USA; 34580). Densitometric analysis was performed using ImageJ (version 1.53t). The following primary antibodies were used for immunoblotting: NLRP3 (#15010), ASC (#13833), cleaved-GSDMD (#36425), cGAS (#15102), p-TBK1 (#5483), TBK1 (#38066), p-STING (#19781), STING (#13647), NF-κB (#8242), Lamin B1 (#13435), and GAPDH (#2118) from Cell Signaling Technology; GSDMD (#20770-1-AP) was from Proteintech (Rosemont, IL, USA).

### 2.3. RNA Preparation and qRT-PCR Analysis

Total RNA was extracted using Easy-Blue reagent (Intron Biotechnology, Seongnam, Gyeonggi-do, Republic of Korea; 17061), and cDNA was synthesized with the QuantiTect Reverse Transcription Kit (Qiagen, Germantown, MD, USA; 205313). Quantitative real-time PCR was conducted using iTaq Universal SYBR Green Supermix (Bio-Rad, Hercules, CA, USA; 1725121) and gene-specific primers (Table 1). Data were analyzed by the 2^−ΔΔCt^ method with normalization to the GAPDH mRNA levels.

### 2.4. Nuclear Fractionation

To evaluate NF-κB translocation, cytoplasmic and nuclear protein fractions were prepared. Cells were lysed in buffer (20 mM HEPES, 10 mM KCl, 1.5 mM MgCl_2_, 1 mM EDTA, 1 mM EGTA, 1 mM DTT, 0.1 mM PMSF, and 250 mM sucrose) and incubated on ice for 30 min. The lysate was sheared through a 32G needle and centrifuged at 1200 rpm for 3 min at 4 °C. The supernatant was collected as the cytosolic fraction. The pellet was washed and lysed in cell lysis buffer, and nuclear proteins were recovered after centrifugation at 15,000 rpm for 15 min at 4 °C.

### 2.5. Confocal Microscopy for Oxidized DNA Detection

Cells were seeded on coverslips in 12-well plates, fixed in 4% paraformaldehyde (Biosesang, Yongin, Gyeonggi-do, South Korea; PC2031-050-00) for 10 min, and permeabilized with 0.01% NP-40 for 5 min. After blocking with 3% BSA (GenDEPOT, Houston, TX, USA; A0100-010) in PBS, cells were incubated with anti-8-OHdG primary antibody (Santa Cruz Biotechnology, Dallas, TX, USA; sc-66036, 1:50) for 48 h at 4 °C. Alexa Fluor 488-conjugated secondary antibodies (Invitrogen, Waltham, MA, USA; A21202, 1:1000) were used for detection. Nuclei were stained with DAPI ( Thermo Scientific, Waltham, MA, USA; 62247), and samples were mounted using ProLong Gold Antifade Reagent (Life Technologies, Waltham, MA, USA; P36930). Imaging was performed using a TCS SP8 confocal microscope (Leica Microsystems, Wetzlar, Germany).

### 2.6. Measurement of Cytosolic mtDNA

Following treatment with rotenone or cyclosporin A (CsA), cells were lysed with 0.01% NP-40 on ice for 20 min. Lysates were centrifuged at 14,000× *g* for 20 min at 4 °C to separate cytosolic fractions. Cytosolic and total DNA were extracted using the NucleoSpin Tissue Mini Kit (Macherey-Nagel, Düren, Germany; 740952.50). qRT-PCR was performed for mitochondrial CYB gene, and cytosolic mtDNA levels were normalized to total cellular mtDNA.

### 2.7. Measurement of IL-1β Secretion

IL-1β levels in the culture supernatant were quantified using a Human IL-1β ELISA Kit (R&D Systems, Minneapolis, MN, USA; DY201) according to the manufacturer’s instructions. Absorbance at 450 nm (reference: 570 nm) was measured using a microplate reader (Molecular Devices, San Jose, CA, USA).

### 2.8. Statistical Analysis

Statistical analyses were performed using GraphPad Prism 7.0 software (GraphPad Software, Inc., San Diego, CA, USA). Data are presented as the mean ± standard deviation (SD), and statistical significance was defined as *p* < 0.05. For comparisons among multiple groups, one-way analysis of variance (ANOVA) followed by Tukey’s post hoc test was used to assess statistical significance.

## 3. Results

### 3.1. Rotenone Activates the NLRP3 Inflammasome Through the cGAS–STING Pathway in THP-1-Derived Macrophages

**Figure 1:**
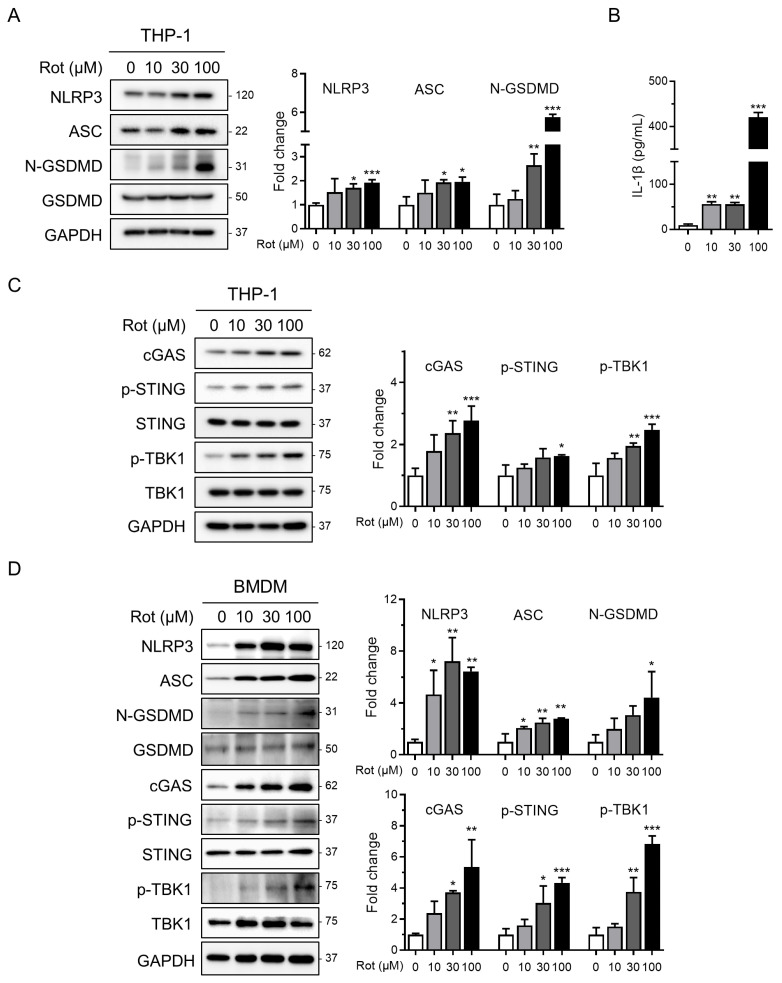
Rotenone activates the NLRP3 inflammasome and cGAS–STING pathway in THP-1 macrophages. THP-1-derived macrophages were treated with rotenone at the indicated concentrations for 6 h. (**A**) Protein expression was analyzed by Western blot. Quantification of band intensities is shown in the right panels. NLRP3 and ASC were normalized to GAPDH, and the N-GSDMD was normalized to total GSDMD. (**B**) IL-1β secretion was measured by ELISA. (**C**) Expression levels of cGAS were normalized to GAPDH, while the p-STING and p-TBK1 levels were normalized to total STING and TBK1. (**D**) BMDMs were treated with rotenone at the indicated concentrations for 6 h, as in the THP-1 cells. Data are presented as the mean ± SD (*n* = 3). Statistical significance was determined by one-way ANOVA followed by Tukey’s post hoc test. * *p* < 0.05, ** *p* < 0.01, *** *p* < 0.001.

To assess NLRP3 inflammasome activation by rotenone, we first evaluated its cytotoxic effects in PMA-differentiated THP-1 macrophages. Cells were treated with increasing concentrations of rotenone for 6 h, and viability was measured using the WST-1 assay. The half-maximal inhibitory concentration (IC_50_) was calculated to be 26.65 µM, with 100 µM reducing viability to approximately 40%. Western blot analysis showed that rotenone dose-dependently increased NLRP3 and ASC protein levels (Figure 1A). In parallel, the ratio of cleaved to pro-GSDMD, indicative of caspase-1 activation, also increased in a dose-dependent manner (Figure 1A). Consistently, IL-1β secretion was markedly elevated approximately fivefold at 10 µM and nearly fortyfold at 100 µM, confirming inflammasome activation (Figure 1B). Notably, qRT-PCR analysis revealed that anti-inflammatory mediators IL-10 and PD-L1 were significantly reduced at 100 µM rotenone, indicating that while pro-inflammatory signaling is strongly induced, counter-regulatory cytokine responses may be suppressed (Figure S1). To investigate the upstream mechanisms, we focused on the cGAS–STING pathway, which senses cytosolic DNA and links mitochondrial stress to inflammatory signaling [27,38]. Rotenone increased the cGAS protein expression and elevated levels of phosphorylated STING and TBK1, indicating activation of this pathway (Figure 1C). These findings were validated in BMDMs, where 30 µM rotenone elicited robust activation of both the cGAS–STING pathway and NLRP3 inflammasome, even more pronounced than in the THP-1 cells (Figure 1D). These results suggest that rotenone activates the NLRP3 inflammasome in THP-1-derived macrophages, potentially via mitochondrial stress and subsequent cGAS–STING pathway activation.

### 3.2. STING Inhibition Attenuates Rotenone-Induced NF-κB–Mediated NLRP3 Priming and Inflammasome Activation

**Figure 2:**
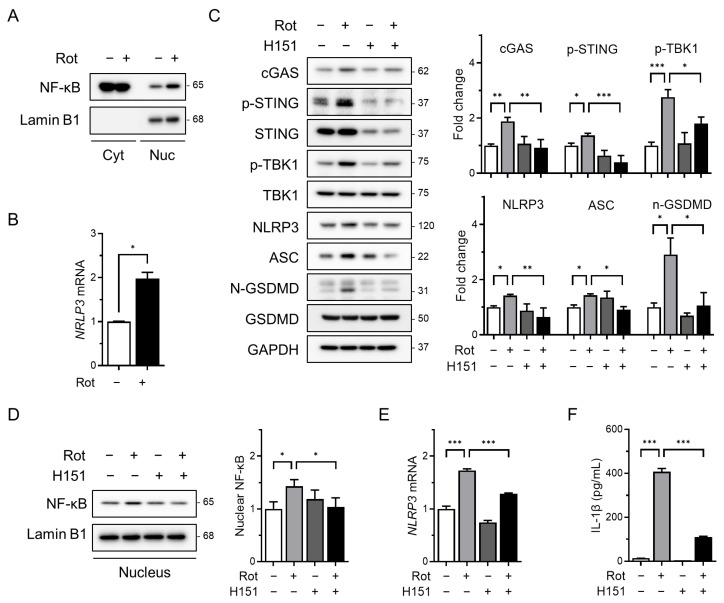
The cGAS–STING pathway mediates priming signals for NLRP3 inflammasome activation. THP-1-derived macrophages were co-treated with rotenone (100 µM) in the presence or absence of the STING inhibitor H151 (10 µM) for 6 h. (**A**) Nuclear fractionation was performed, and nuclear NF-κB levels were analyzed by Western blotting. Lamin B1 was used as a nuclear loading control. (**B**) NLRP3 mRNA expression was analyzed by qRT-PCR and normalized to GAPDH. (**C**) Protein levels of cGAS, p-STING, NLRP3, ASC, p-TBK1, and GSDMD were analyzed by Western blotting. Quantification of band intensities is shown in the right panels. (**D**) Nuclear NF-κB levels were assessed by Western blotting using nuclear extracts. Lamin B1 was used as a nuclear loading control. (**E**) NLRP3 mRNA expression was quantified by qRT-PCR and normalized to GAPDH. (**F**) IL-1β secretion was measured by ELISA. Data are presented as the mean ± SD (*n* = 3). Statistical significance was determined by one-way ANOVA followed by Tukey’s post hoc test. * *p* < 0.05, ** *p* < 0.01, *** *p* < 0.001.

As the cGAS–STING pathway is known to activate NF-κB signaling in response to cytosolic double-stranded DNA [19,25], we investigated whether rotenone affects the transcriptional priming of the NLRP3 inflammasome. Rotenone treatment significantly increased the nuclear NF-κB levels, as determined by Western blotting of the nuclear and cytoplasmic fractions (Figure 2A). This was accompanied by a marked upregulation of NLRP3 mRNA (Figure 2B), indicating enhanced priming of the inflammasome.

To assess the role of STING in this process, we co-treated cells with rotenone and H151, a selective and irreversible STING inhibitor that blocks cGAMP-induced STING oligomerization [39]. H151 effectively suppressed rotenone-induced upregulation of cGAS, phosphorylated STING, and phosphorylated TBK1 (Figure 2C). This was associated with decreased levels of NLRP3 and ASC as well as a reduced cleaved-GSDMD/GSDMD ratio (Figure 2C). Additionally, H151 treatment reduced the nuclear NF-κB accumulation and NLRP3 mRNA expression (Figure 2D,E), indicating that cGAS–STING signaling contributes to NF-κB–mediated transcriptional priming of the NLRP3 inflammasome. Consistent with these findings, ELISA revealed that IL-1β secretion was also markedly reduced following H151 treatment (Figure 2F), further supporting the attenuation of inflammasome activity.

### 3.3. Mitochondrial ROS–mPTP Axis Promotes cGAS–STING-Mediated NLRP3 Inflammasome Activation

**Figure 3:**
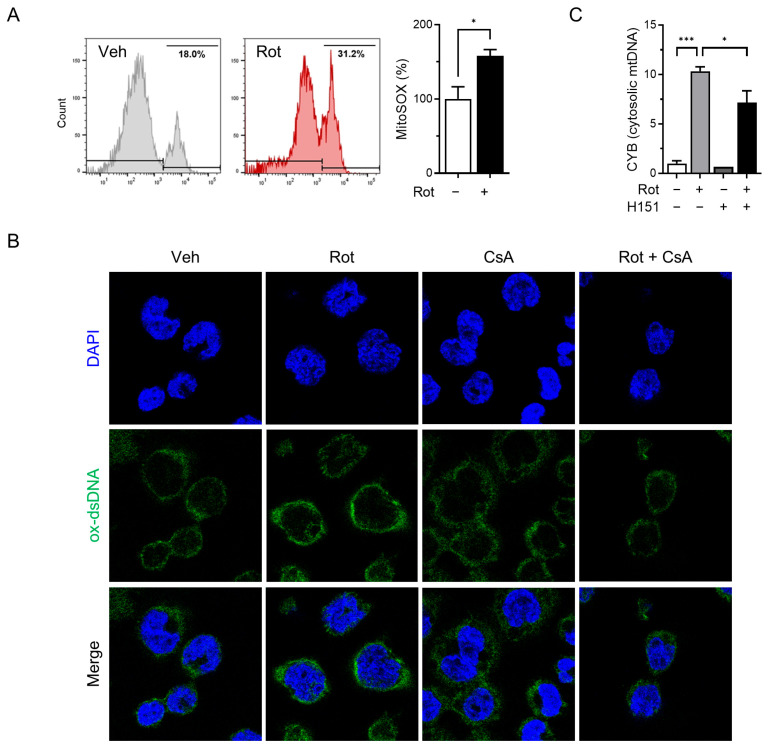
Rotenone induces the release of oxidized mitochondrial DNA. THP-1-derived macrophages were co-treated with rotenone (100 µM) in the presence or absence of cyclosporin A (CsA, 10 µM) for 6 h. (**A**) Mitochondrial ROS were detected using MitoSOX fluorescence. (**B**) Immunofluorescence staining was performed using an antibody against oxidized double-stranded DNA (ox-dsDNA; green). Nuclei were counterstained with DAPI (blue). (**C**) qRT-PCR was used to quantify the mtDNA (CYB gene) levels in cytosolic and total cell lysates. Cytosolic mtDNA levels were normalized to total mtDNA levels. Data are presented as the mean ± SD (*n* = 3). Statistical significance was determined by one-way ANOVA followed by Tukey’s post hoc test. * *p* < 0.05, *** *p* < 0.001.

Mitochondrial dysfunction and elevated ROS levels are known to trigger opening of the mPTP, facilitating the release of mtDNA into the cytosol [40]. Previous studies have demonstrated that rotenone induces mitochondrial dysfunction and mPTP opening in liver cells [41], and increases cytosolic mtDNA levels in BMDMs [35]. To determine whether rotenone induces similar mitochondrial stress in THP-1-derived macrophages, we measured the mitochondrial ROS using MitoSOX and flow cytometry. Treatment with 100 µM rotenone resulted in a 1.5-fold increase in mitochondrial ROS compared with the control (Figure 3A).

**Figure 4:**
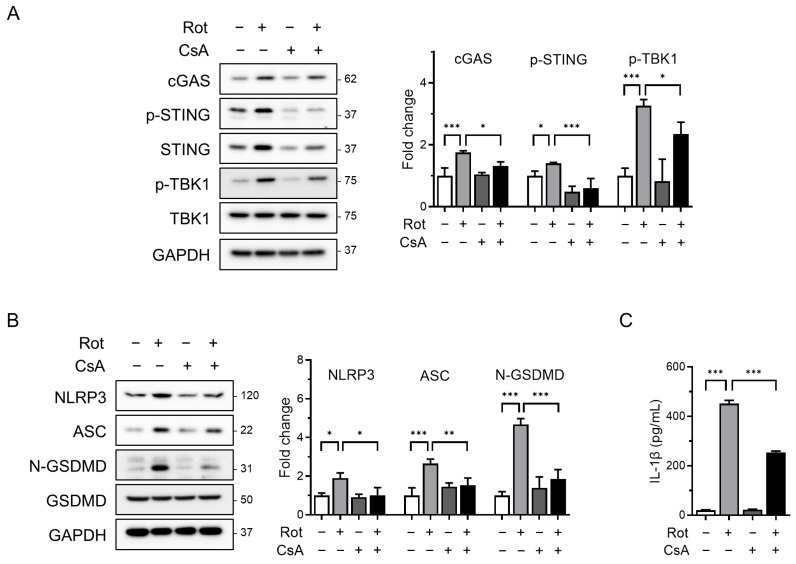
Blocking mPTP opening attenuates NLRP3 inflammasome activation and cGAS–STING signaling. THP-1-derived macrophages were co-treated with rotenone (100 µM) in the presence or absence of cyclosporin A (CsA, 10 µM) for 6 h. (**A**,**B**) Protein levels were analyzed by Western blot. Quantification of band intensities is shown in the right panels. cGAS, NLRP3, and ASC were normalized to GAPDH; p-STING was normalized to total STING; p-TBK1 was normalized to total TBK1; and N-GSDMD was normalized to total GSDMD. (**C**) IL-1β secretion was quantified by ELISA. Data are presented as the mean ± SD (*n* = 3). Statistical significance was determined by one-way ANOVA followed by Tukey’s post hoc test. * *p* < 0.05, ** *p* < 0.01, *** *p* < 0.001.

**Figure 5:**
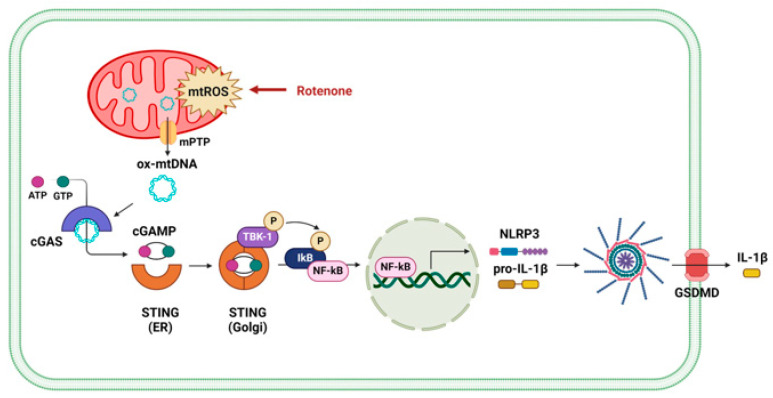
Schematic illustration of the proposed mechanism by which rotenone activates the NLRP3 inflammasome via mPTP opening and mtDNA-mediated cGAS–STING signaling.

To determine whether mitochondrial ROS contributes to the release of oxidized mtDNA and subsequent NLRP3 inflammasome activation, we pretreated cells with cyclosporin A (CsA), a cyclophilin D inhibitor that prevents mPTP opening, prior to rotenone exposure. Confocal microscopy revealed substantial accumulation of cytoplasmic oxidized double-stranded DNA (ox-dsDNA) following rotenone treatment, which was significantly reduced by CsA co-treatment (Figure 3B). Furthermore, qRT-PCR analysis showed that the cytosolic mtDNA levels (CYB gene) were elevated by rotenone and reduced upon CsA treatment, supporting a link between mitochondrial stress, mtDNA release, and cGAS activation (Figure 3C). Western blotting demonstrated that CsA suppressed the rotenone-induced activation of cGAS, phosphorylated STING, and TBK1 (Figure 4A). This was accompanied by decreased NLRP3 and ASC expression, reduced cleaved-GSDMD levels, and markedly lower IL-1β secretion (Figure 4B,C). Similar results were obtained in the CsA-treated BMDMs, confirming that the inhibition of mPTP prevents rotenone-induced cGAS–STING and NLRP3 inflammasome activation in a primary macrophage model (Figure S2). These findings indicate that rotenone-induced mitochondrial ROS promotes mPTP opening, facilitating the cytosolic release of oxidized mtDNA. This, in turn, activates the cGAS–STING pathway, contributing to NLRP3 inflammasome activation (Figure 5). Collectively, our results provide mechanistic insight into how mitochondrial dysfunction is linked to inflammatory signaling via the cGAS–STING–NLRP3 axis.

## 4. Discussion

Mitochondrial dysfunction is increasingly recognized as a key driver of innate immune activation. However, the precise molecular mechanisms linking mitochondrial stress to NLRP3 inflammasome assembly remain incompletely defined. In this study, we identified the cGAS–STING pathway as a critical mediator of NLRP3 inflammasome activation in rotenone-treated THP-1-derived macrophages.

Unlike classical NLRP3 activators, which typically require separate priming (e.g., LPS) and activation (e.g., ATP) signals, rotenone alone is sufficient to induce both phases of inflammasome activation. This observation aligns with recent evidence that mitochondrial DAMPs can bypass canonical priming requirements by activating both transcriptional and post-translational pathways of inflammasome signaling [25,26,27]. Historically, rotenone has been studied in the context of neurodegenerative diseases, particularly Parkinson’s disease, where mitochondrial complex I inhibition and neuroinflammation are prominent pathological features [35,36,42]. Previous studies have demonstrated that rotenone could facilitate either the priming or activation of the NLRP3 inflammasome, but typically required co-treatment with LPS or secondary stimuli to achieve full inflammasome activation [35,36]. In contrast, our data showed that rotenone alone robustly activates the NLRP3 inflammasome, mediated by mitochondrial ROS production, mPTP opening, and the subsequent cytosolic release of oxidized mtDNA.

Mechanistically, we demonstrated that released mtDNA triggers the cGAS–STING pathway, which in turn promotes NF-κB nuclear translocation and the transcriptional upregulation of NLRP3. We assessed cytosolic mtDNA release by quantifying the mitochondrial-encoded CYB gene, which increased significantly after rotenone treatment, providing direct evidence of mtDNA leakage. While CYB served as a representative marker, previous studies indicate that multiple mtDNA regions (e.g., ND1, ND4, COX1) can also be released under mitochondrial stress [43]. Future work will expand this analysis to better define the composition of released mtDNA. These findings align with established roles of cGAS–STING in mediating type I interferon and NF-κB responses [44,45,46,47], and support reports that cGAS–STING promotes NLRP3 inflammasome assembly by enhancing ER localization and reducing polyubiquitination [25,48,49]. Pharmacological inhibition further supports the involvement of this pathway: H151, a selective STING inhibitor that blocks palmitoylation and STING clustering [39], significantly reduced NLRP3 expression, IL-1β secretion, and caspase-1 activation. Likewise, cyclosporin A, which inhibits mPTP opening by targeting cyclophilin D [50], suppressed mtDNA release and downstream inflammasome signaling. Classical antioxidants, such as N-acetylcysteine, can scavenge mitochondrial ROS, thereby reducing mtDNA release and indirectly limiting cGAS–STING activation. However, unlike CsA, antioxidants do not directly prevent mPTP opening, highlighting the complementary roles of ROS scavengers and pore inhibitors in modulating this inflammatory axis [51,52]. Notably, cGAS–STING activation also influences broader cytokine responses. While we focused on pro-inflammatory cytokines such as IL-1β, this pathway can induce type I interferons and may modulate anti-inflammatory mediators such as IL-10 and PD-L1, representing a counter-regulatory mechanism to restrain inflammation and restore homeostasis [53,54]. Together, these findings establish the mitochondrial ROS–mPTP–cGAS–STING axis as a central regulator of NLRP3 inflammasome activation.

Therapeutically, these insights demonstrate that targeting this axis could be a promising strategy for treating sterile inflammatory diseases including neurodegenerative disorders and inflammatory lung injury [55,56]. However, systemic inhibition of STING may compromise host antiviral immunity, as STING plays an essential role in sensing cytosolic viral DNA and coordinating antiviral responses [57]. These concerns underscore the importance of cell type-specific or context-dependent strategies when targeting STING therapeutically. Our findings also expand the utility of rotenone beyond its traditional role as a neurotoxin in Parkinson’s disease models. Given its widespread use as an agricultural pesticide, chronic environmental exposure to rotenone may contribute to inflammatory pathologies in peripheral organs such as the liver and kidney. Notably, the cGAS–STING pathway has been implicated in metabolic syndrome and chronic kidney disease [58], suggesting broader relevance for our findings. Moreover, our data reinforce the emerging view that oxidized mtDNA serves as a universal sentinel of cellular stress, capable of activating both NLRP3 inflammasome assembly and type I interferon signaling via cGAS–STING.

Several limitations should be acknowledged. First, our experiments were conducted exclusively in THP-1-derived macrophages, which are widely used as a standard model for studying NLRP3 inflammasome biology [59] due to their robust innate immune signaling and experimental tractability. However, THP-1 cells cannot fully recapitulate the complexity of primary human macrophages, and validation of our findings in primary macrophages will therefore be an important priority for future studies. It also remains to be determined whether similar cGAS–STING-dependent mechanisms operate in non-immune cells such as neurons or hepatocytes. While rotenone has been shown to induce mitochondrial dysfunction and apoptosis in hepatocyte cell lines (e.g., HepG2, WB-F344) [41,60], whether this leads to cGAS–STING-mediated inflammasome activation in these cells is unclear. Evidence from neuronal and other non-immune models suggests that mtDNA release and cGAS–STING signaling can occur with cell type-specific outcomes: neurons may exhibit neuroinflammation via cGAS–STING–NLRP3 activation [42,48,61], hepatocytes primarily induce type I interferon and autophagy responses rather than robust inflammasome assembly [62], and Kupffer cells are predicted to mount strong pro-inflammatory responses through cGAS–STING–NLRP3 activation upon rotenone exposure [63]. Second, while we focused on the cGAS–STING pathway, other cytosolic DNA sensors, including TLR9 and AIM2, may also contribute to rotenone-induced inflammation. Oxidized mtDNA can engage multiple pattern recognition receptors, suggesting that distinct pathways may be activated depending on the stimulus and cell type. Future studies utilizing gene knockouts or pharmacological inhibitors could help elucidate the contributions of these alternative DNA-sensing mechanisms. Finally, rotenone is known to impair mitophagy, raising the possibility that the defective clearance of damaged mitochondria contributes to sustained mtDNA release and chronic inflammation. Previous studies have shown that rotenone disrupts mitophagy via CK2 activation in neuronal cells [42,64], supporting a feed-forward loop in which impaired mitochondrial quality control exacerbates inflammasome signaling. The interaction between mitophagy and inflammasome activation represents a critical area for future investigation.

In conclusion, our findings establish a mechanistic framework in which mitochondrial dysfunction and mtDNA release activate the cGAS–STING pathway, thereby promoting both priming and assembly of the NLRP3 inflammasome in the absence of classical stimuli. This work advances our understanding of how mitochondrial stress interfaces with innate immune signaling and identifies actionable targets within the mtROS–mPTP–cGAS–STING axis for treating inflammatory diseases triggered by environmental toxins or metabolic stressors.

## Figures and Tables

**Table 1:** List of Primers for qRT-PCR.

Gene	Forward Primer (5′ to 3′)	Reverse Primer (5′ to 3′)
*NLRP3*	GGACTGAAGCACCTGTTGTGCA	TCCTGAGTCTCCCAAGGCATTC
*GAPDH*	AACGGATTTGGTCGTATTG	GCTCCTGGAAGATGGTGAT
*MT-CYB*	ATCACTCGAGACGTAAATTATGGCT	TGAACTAGGTCTGTCCCAATGTATG

## Data Availability

The data presented in this study are available on request from the corresponding author.

## Supplementary Materials

The following supporting information can be downloaded at: https://www.mdpi.com/article/10.3390/antiox14111276/s1, Figure S1: Rotenone decreases anti-inflammatory cytokines in THP-1 macrophages; Figure S2: Blocking mPTP opening attenuates NLRP3 inflammasome activation and cGAS–STING signaling.
