# Supplementary material for: cGAS–STING–NF-κB Axis Mediates Rotenone-Induced NLRP3 Inflammasome Activation Through Mitochondrial DNA Release
Source: Antioxidants (Basel). 2025 Oct 24;14(11):1276. doi: 10.3390/antiox14111276 (PMC12649239; doi:10.3390/antiox14111276)
Supplement: Supplementary file 1 [file antioxidants-14-01276-s001.zip › antioxidants-3798957-supplementary.pdf]

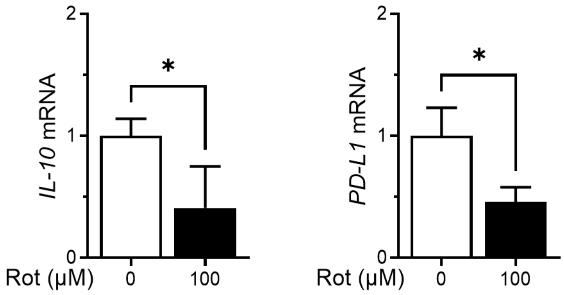

Figure S1. Rotenone decreases anti-inflammatory cytokines in THP-1 macrophages. THP-1-derived macrophages were treated with 100 μM rotenone for 6 h. mRNA levels of IL-10 and PD-L1 were measured by qRT-PCR. Data are presented as mean ± SD (n = 3). Statistical significance was determined by Student's t-test. *P < 0.05.

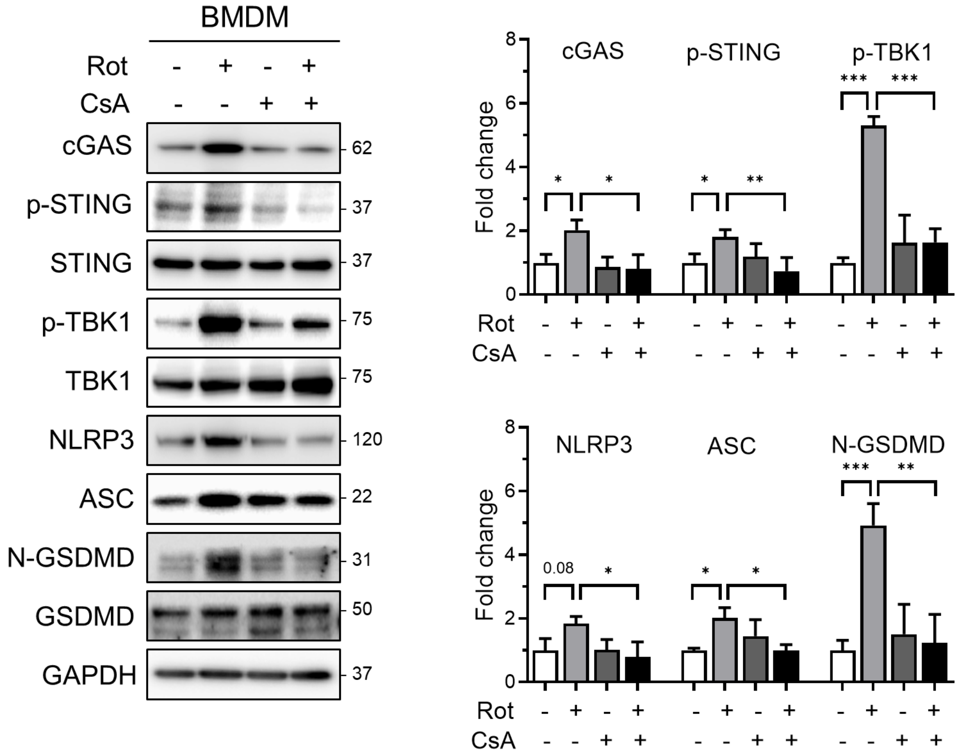

Figure S2. Blocking mPTP opening attenuates NLRP3 inflammasome activation and cGAS–STING signaling. BMDMs were co-treated with 30 μM rotenone in the presence or absence of cyclosporin A (CsA, 10 μM) for 6 h. Protein levels were analyzed by western blot, and quantification of band intensities is shown in the right panels. cGAS, NLRP3, and ASC were normalized to GAPDH; p-STING was normalized to total STING; p-TBK1 was normalized to total TBK1; and N-GSDMD was normalized to total GSDMD. Data are presented as mean ± SD (n = 3). Statistical significance was determined by one-way ANOVA followed by Tukey's post hoc test. *P < 0.05, **P < 0.01, ***P < 0.001.
